# SARS-CoV-2 Proteins Exploit Host’s Genetic and Epigenetic Mediators for the Annexation of Key Host Signaling Pathways

**DOI:** 10.3389/fmolb.2020.598583

**Published:** 2021-01-27

**Authors:** Md. Abdullah-Al-Kamran Khan, Abul Bashar Mir Md. Khademul Islam

**Affiliations:** ^1^ Department of Mathematics and Natural Sciences, BRAC University, Dhaka, Bangladesh; ^2^ Department of Genetic Engineering and Biotechnology, University of Dhaka, Dhaka, Bangladesh

**Keywords:** host-virus interactions, COVID-19, SARS-CoV-2, immune evasion, epigenetic regulation, host immune response

## Abstract

The constant rise of the death toll and cases of COVID-19 has made this pandemic a serious threat to human civilization. Understanding of host-SARS-CoV-2 interaction in viral pathogenesis is still in its infancy. In this study, we utilized a blend of computational and knowledgebase approaches to model the putative virus-host interplay in host signaling pathways by integrating the experimentally validated host interactome proteins and differentially expressed host genes in SARS-CoV-2 infection. While searching for the pathways in which viral proteins interact with host proteins, we discovered various antiviral immune response pathways such as hypoxia-inducible factor 1 (HIF-1) signaling, autophagy, retinoic acid-inducible gene I (RIG-I) signaling, Toll-like receptor signaling, fatty acid oxidation/degradation, and IL-17 signaling. All these pathways can be either hijacked or suppressed by the viral proteins, leading to improved viral survival and life cycle. Aberration in pathways such as HIF-1 signaling and relaxin signaling in the lungs suggests the pathogenic lung pathophysiology in COVID-19. From enrichment analysis, it was evident that the deregulated genes in SARS-CoV-2 infection might also be involved in heart development, kidney development, and AGE-RAGE signaling pathway in diabetic complications. Anomalies in these pathways might suggest the increased vulnerability of COVID-19 patients with comorbidities. Moreover, we noticed several presumed infection-induced differentially expressed transcription factors and epigenetic factors, such as miRNAs and several histone modifiers, which can modulate different immune signaling pathways, helping both host and virus. Our modeling suggests that SARS-CoV-2 integrates its proteins in different immune signaling pathways and other cellular signaling pathways for developing efficient immune evasion mechanisms while leading the host to a more complicated disease condition. Our findings would help in designing more targeted therapeutic interventions against SARS-CoV-2.

## Introduction

Though several human coronavirus outbreaks caused severe public health crises over the past few decades, the recent coronavirus disease (COVID-19) outbreak caused by the severe acute respiratory syndrome coronavirus 2 (SARS-CoV-2) has beaten the records of the previous ones and the case counts are still on the upswing. About 210 countries and territories around the globe have been affected by this outbreak, ∼24 million people are already infected with SARS-CoV-2, and the number was steadily rising at the time of writing this article (Worldometer, 2020). Out of the closed cases, almost 7% of the patients have died and about 1% of the active cases are in critical conditions (Worldometer, 2020). Though the death rates from COVID-19 were estimated to be as low as 3.4% (WHO, 2020), at present, the global fatality rate is changing very rapidly; therefore, more comprehensive studies need to be conducted to effectively control and overturn this pandemic.

Coronaviruses are single-stranded positive-sense, enveloped RNA viruses having ∼30 Kb genome (Lu et al., 2020). Among the four genera, SARS-CoV-2 (accession no. NC_045512.2) belongs to the *Betacoronavirus* genus and it has ∼29.9 Kb genome encoding 11 genes (NCBI-Gene, 2020). The genome sequence of SARS-CoV-2 is about 90% similar to bat-derived SARS-like coronavirus, whereas this novel virus is only ∼79 and ∼50% similar to severe acute respiratory syndrome coronavirus (SARS-CoV) and Middle East Respiratory Syndrome-related Coronavirus (MERS-CoV), respectively (Lu et al., 2020; Ren et al., 2020). A substantial genomic difference can be observed between SARS-CoV and SARS-CoV-2; as in SARS-CoV-2, there have been 380 amino acids substitution, deletion of ORF8a, elongation of ORF8b, and truncation of ORF3b observed (Lu et al., 2020).

Though the overall mortality rate from SARS-CoV is higher than that of SARS-CoV-2, several unique features of SARS-CoV-2, including increased incubation period and dormancy inside the host, enabled this virus to spread more efficiently (Lauer et al., 2020). This suggests that SARS-CoV-2 might be using some immune evasion strategies to maintain its survival and essential functions within the host.

Upon viral infection, the host innate immune system detects the virion particles and elicits the first sets of antiviral responses (Katze et al., 2008) to eliminate the viral threats. However, viruses themselves have generated various modes of action to evade those immune responses by modulating the host’s intracellular signaling pathways (Kikkert, 2020). This arm-wrestling between the host and the infecting virus results in immunopathogenesis. Different human coronaviruses also show similar features of host-pathogen interactions, which range from the viral entry, replication, transcription, translation, and assembly to the evasion from host innate immune response (Fung and Liu, 2019). Moreover, different antiviral cellular responses such as autophagy (Ahmad et al., 2018) and apoptosis (Barber, 2001) can also be moderated by the virus to ensure its survival inside the host cells. Apart from these, several other host-virus interactions are also observed, namely, modulation of the activity of host transcription factors (TFs) (Lyles, 2000) and host epigenetic factors (e.g., histone modifications and host miRNAs) (Adhya and Basu, 2010). All of these multifaceted interactions can lead to the ultimate pathogenesis and progression of the disease.

The interplay between different human coronaviruses and host was previously reported (Fung and Liu, 2019); however, SARS-CoV-2 interactions with the host immune response and its outcome in the pathogenesis are yet to be elucidated. Gordon et al. (2020) identified 332 high-confidence interactions between SARS-CoV-2 proteins and human proteins (Gordon et al., 2020); Blanco-Melo et al. (2020) produced transcriptional signatures of SARS-CoV-2 infected cells (Blanco-Melo et al., 2020). In this study, we aimed to model the complex host-SARS-CoV-2 interactions with the associated differentially expressed genes found in the SARS-CoV-2 infection to gain insights into the probable immune escape mechanisms of SARS-CoV-2. Also, we have compared the differential gene expression profiles of SARS-CoV and SARS-CoV-2 infected cells to find out the pathways uniquely targeted by SARS-CoV-2. Moreover, we incorporated other associated host epigenetic factors that might play a role in the pathogenesis by deregulating the signaling pathways.

## Materials and Methods

### Retrieval of the Host Proteins That Interact With SARS-CoV-2

We have obtained 332 human proteins that form high-confidence interactions with SARS-CoV-2 proteins from the study conducted previously by Gordon et al. (2020) and processed their provided protein names into the associated HGNC official gene symbol (Supplementary Material S1).

### Analysis of Microarray Expression Data

Microarray expression data on SARS-CoV infected 2B4 cells or uninfected controls for 24 h were obtained from Gene Expression Omnibus (GEO) (https://www.ncbi.nlm.nih.gov/geo) (Barrett et al., 2012), accession: GSE17400 (Yoshikawa et al., 2010). Raw Affymetrix CEL files were background-corrected and normalized using Bioconductor package “affy v1.28.1” using “rma” algorithm. Quality of microarray experiment (data not shown) was verified by Bioconductor package “arrayQualityMetrics v3.2.4” (Kauffmann et al., 2009). Differential expression (DE) between two experimental conditions was detected using Bioconductor package Limma (Smyth, 2005). Probe annotations were converted to genes using in-house python script basing the Ensembl gene model (BioMart 99) (Flicek et al., 2007). The highest absolute expression value was considered for the probes that were annotated to the same gene. We have considered the genes to be differentially expressed, which have false discovery rate (FDR) (Benjamini and Hochberg, 1995) *p* value ≤0.05 and Log_2_ fold change value ≥0.25. Positive Log_2_ fold change values indicate upregulation, whereas negative ones indicate downregulation.

### Analysis of RNA-Seq Expression Data

Illumina sequenced RNA-Seq raw FastQ reads were extracted from the GEO database (https://www.ncbi.nlm.nih.gov/geo) (Barrett et al., 2012), accession: GSE147507 (Blanco-Melo et al., 2020). These data included independent biological triplicates of primary human lung epithelium (NHBE) that were mock-treated or infected with SARS-CoV-2 for 24 h. Mapping of reads was done with TopHat v2.1.1 (with Bowtie v2.4.1) (Trapnell et al., 2009). Short reads were uniquely aligned, allowing at best two mismatches to the human reference genome from GRCh38 as downloaded from the USCS database (Lander et al., 2001). Sequences matched exactly at more than one place with equal quality were discarded to avoid bias (Hansen et al., 2010). The reads that were not mapped to the genome were utilized to map against the transcriptome (junction mapping). Ensembl gene model (Hubbard et al., 2007) (version 99, as extracted from UCSC) was used for this process. After mapping, we used SubRead package featureCount v2.21 (Liao et al., 2013) to calculate absolute read abundance (read count, rc) for each transcript/gene associated with the Ensembl genes. For DE analysis, we used DESeq2 v1.26.0 with R v3.6.2 (2019-07-05) (Anders and Huber, 2010) that uses a model based on the negative binomial distribution. To avoid false positives, we considered only those transcripts where at least 10 reads are annotated in at least one of the samples used in this study. We have considered the genes to be differentially expressed when they have FDR (Benjamini and Hochberg, 1995) *p* value ≤0.05 and Log2 fold change value ≥0.25. Positive Log_2_ fold change values mean upregulation, whereas negative ones mean downregulation.

### Functional Enrichment Analysis

We utilized Gitools v1.8.4 for enrichment analysis and heatmap generation (Perez-Llamas and Lopez-Bigas, 2011). We have utilized the Gene Ontology Biological Processes (GOBP) and Molecular Function (GOMF) modules (Ashburner et al., 2000), KEGG Pathways (Kanehisa and Goto, 2000), BioPlanet pathways (Huang et al., 2019b) modules, and WikiPathways (Slenter et al., 2017) modules for the overrepresentation analysis. Resulting *p*-values were adjusted for multiple testing using the Benjamini–Hochberg method of FDR (Benjamini and Hochberg, 1995). We have also performed the enrichment analysis based on the KEGG pathway module of the STRING database (Szklarczyk et al., 2019) for 332 proteins (Supplementary Material S1) retrieved from the analysis of Gordon et al. (2020) (Gordon et al., 2020) along with the deregulated genes analyzed from SARS-CoV-2 infected cell’s RNA-Seq expression data. An enrichment is considered significant if its adjusted *p* value is <0.05.

### Obtaining the Transcription Factors That Bind Promoter Regions

We have obtained the TFs that bind to the given promoters from the Cistrome Data Browser (Zheng et al., 2018), which provides TFs from experimental ChIP-seq data. We utilized “Toolkit for CistromeDB,” uploaded the 5 Kb upstream promoter with 1 Kb downstream from transcription start site (TSS) BED file of the deregulated genes, and fixed the peak number parameter to “all peaks in each sample.”

### Obtaining Human miRNAs Target Genes

We extracted the experimentally validated target genes of human miRNAs from miRTarBase database (Huang et al., 2019a). We only considered those miRNAs whose target gene was found downregulated and the associated TF upregulated in the differential gene expression analysis.

### Extraction of Transcription Factors That Modulate Human miRNA Expression

We have downloaded the experimentally validated TFs from the TransmiR v2.0 database (Tong et al., 2019), which bind to miRNA promoters and modulate them. We have considered those TFs that are expressed themselves and that can “activate” or “regulate” miRNAs.

### Identification of the Host Epigenetic Factors Genes

We used EpiFactors database (Medvedeva et al., 2015) to find human genes related to the epigenetic activity. We only considered those epigenetic factors for the final modeling that are differentially expressed in our analyzed transcriptome of SARS-CoV and SARS-CoV-2.

### Mapping of the Human Proteins in Cellular Pathways

We have utilized the KEGG mapper tool (Kanehisa and Sato, 2020) for the mapping of deregulated genes SARS-CoV-2 interacting host proteins in different cellular pathways. We then searched and targeted the pathways found to be enriched for SARS-CoV-2 deregulated genes. From this pathway information, we have manually sketched the pathways to provide a brief overview of the interplay between SARS-CoV-2 and host immune response and their outcomes in the viral pathogenesis.

**Figure 1:**
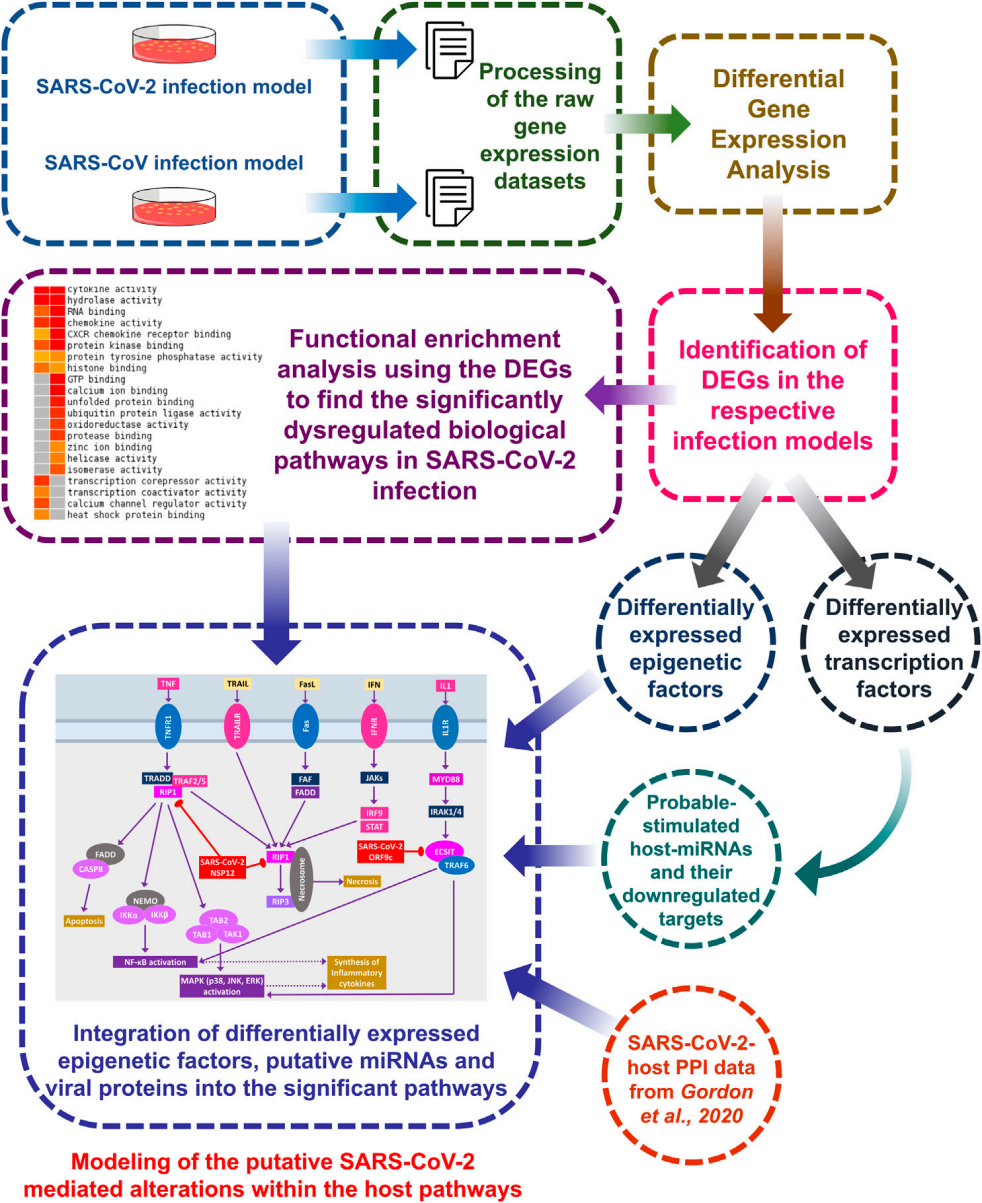
Overall workflow of the study.

A brief workflow of the whole study is depicted in Figure 1.

## Results

### Differentially Expressed Genes in SARS-CoV-2 Infection Are Involved in Important Cellular Signaling Pathways

We wanted to identify the pathways that might be modulated upon the infection of SARS-CoV-2 and to highlight their uniqueness from SARS-CoV infection. Therefore, we performed the enrichment analysis using the differentially expressed genes of both SARS-CoV and SARS-CoV-2 by Gitools (Perez-Llamas and Lopez-Bigas, 2011) using GOBP, GOMF, KEGG pathways, BioPlanet pathways, and WikiPathways modules.

**Figure 2:**
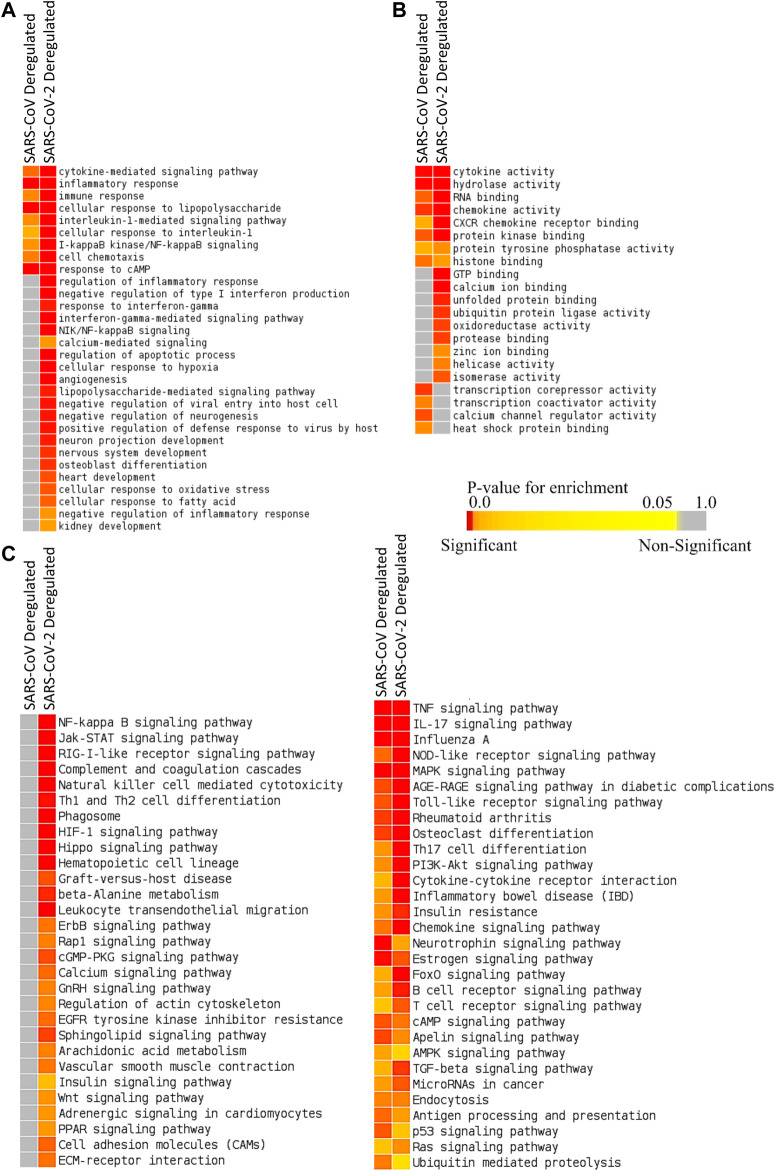
Enrichment analysis and comparison between deregulated genes in SARS-CoV and SARS-CoV-2 infections using **(A)** GOBP module, **(B)** GOMF module, and **(C)** KEGG pathway module. The significance of enrichment in terms of the adjusted *p* value (<0.05) is represented in color-coded *p* value scale for all heatmaps. Color toward red indicates higher significance and color toward yellow indicates less significance, while gray means nonsignificant.

We identified 387 upregulated and 61 downregulated genes in SARS-CoV infection (analyzing GSE17400) and 464 upregulated and 222 downregulated genes in SARS-CoV-2 infection (analyzing GSE147507) (Supplementary Material S2). Enrichment analysis using these differentially expressed genes exhibited that deregulated genes of SARS-CoV-2 infection can exert biological functions such as regulation of inflammatory response, negative regulation of type I interferon, response to interferon-gamma, interferon-gamma-mediated signaling, NIK/NF-kappaB signaling, regulation of the apoptotic process, cellular response to hypoxia, angiogenesis, negative regulation of inflammatory response, zinc ion binding, and calcium ion binding; all of these were not enriched for SARS-CoV infection (Figures 2A,B). Also, different organ-specific functions such as heart development and kidney development were only enriched for differentially expressed genes in SARS-CoV-2 infection (Figure 2A).

Deregulated genes of SARS-CoV-2 infection were involved in pathways such as NF-kappaB signaling, Jak-STAT signaling, RIG-I-like receptor signaling, natural killer cell–mediated cytotoxicity, phagosome, HIF-1 signaling, calcium signaling, GnRH signaling, arachidonic acid metabolism, insulin signaling, adrenergic signaling in cardiomyocytes, and PPAR signaling (Figure 2C; Supplementary Figures S1, S2), which were absent in SARS-CoV infection. These significant differences between these two infections might be crucial in defining the differences in disease pathobiology between these viruses; however, these variations could have been resulted due to the differences in the used infection models.

### Both SARS-CoV-2 Interacting Human Proteins and Deregulated Genes from SARS-CoV-2 Infection Have Immunological Roles

To illuminate whether SARS-CoV-2 interacting human proteins and differentially expressed genes in SARS-CoV-2 infection are involved in the same pathways, we sought out an enrichment analysis using STRING (KEGG pathway module) (Szklarczyk et al., 2019), which is a functional protein-protein interaction network database.

**Figure 3:**
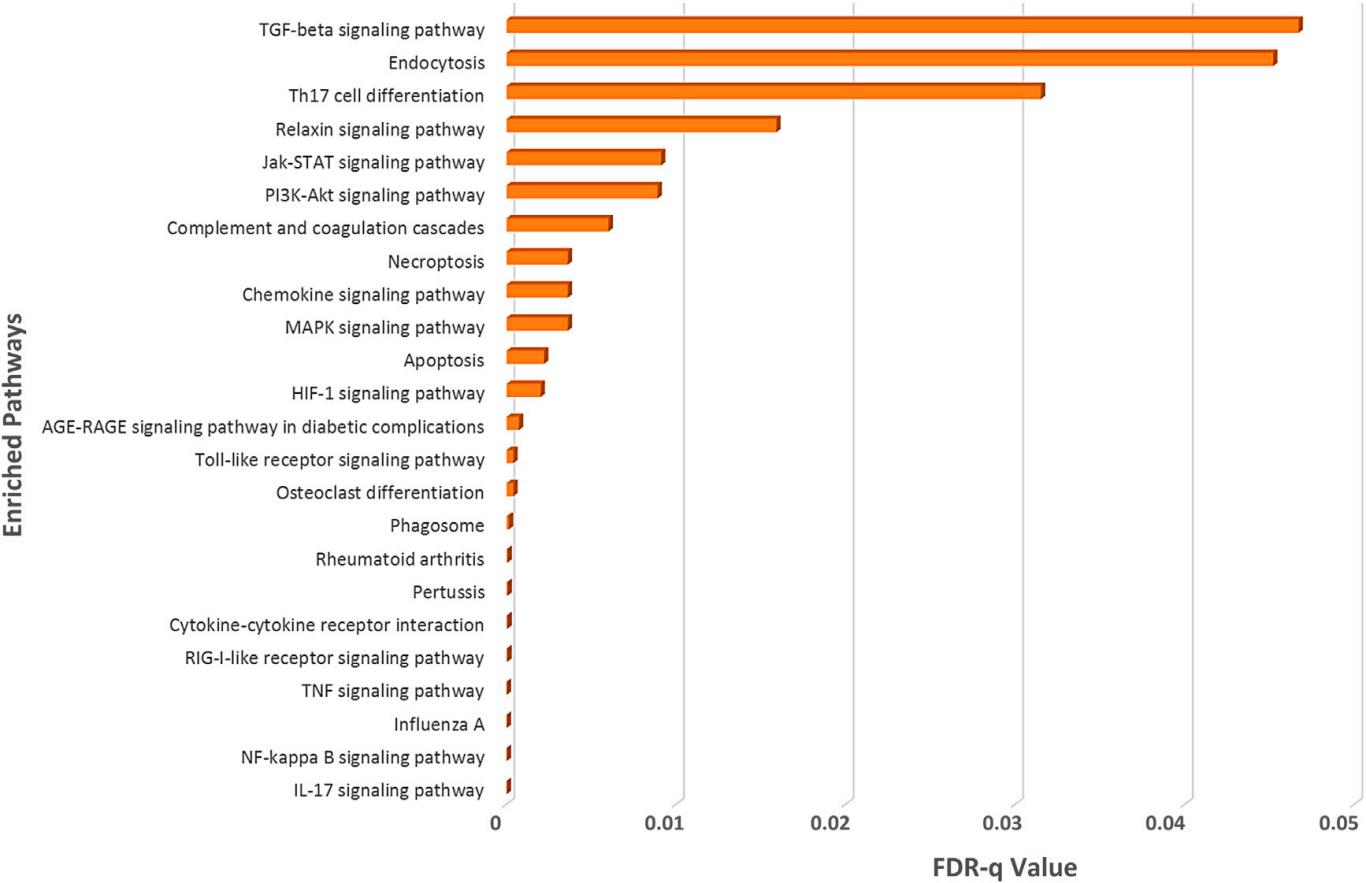
Enrichment analysis of deregulated genes in SARS-CoV-2 infections and human proteins interacting with SARS-CoV-2 proteins using KEGG pathway module of the STRING database.

From this analysis, we spotted that both the deregulated genes of SARS-CoV-2-infection and the SARS-CoV-2-interacting human proteins are involved in several important immune signaling pathways, namely, IL-17 signaling, NF-kappaB signaling, TNF signaling, Toll-like receptor signaling, phagosome, apoptosis, necroptosis, PI3K-Akt signaling, HIF-1 signaling, and MAPK signaling (Figure 3). Moreover, signaling pathways such as relaxin signaling, rheumatoid arthritis, and AGE-RAGE signaling pathway in diabetic complications were also enriched (Figure 3).

### Differentially Expressed Genes in SARS-CoV-2 Infection Have Different Transcription Factor Binding Preferences Compared to SARS-CoV Infection

**Figure 4:**
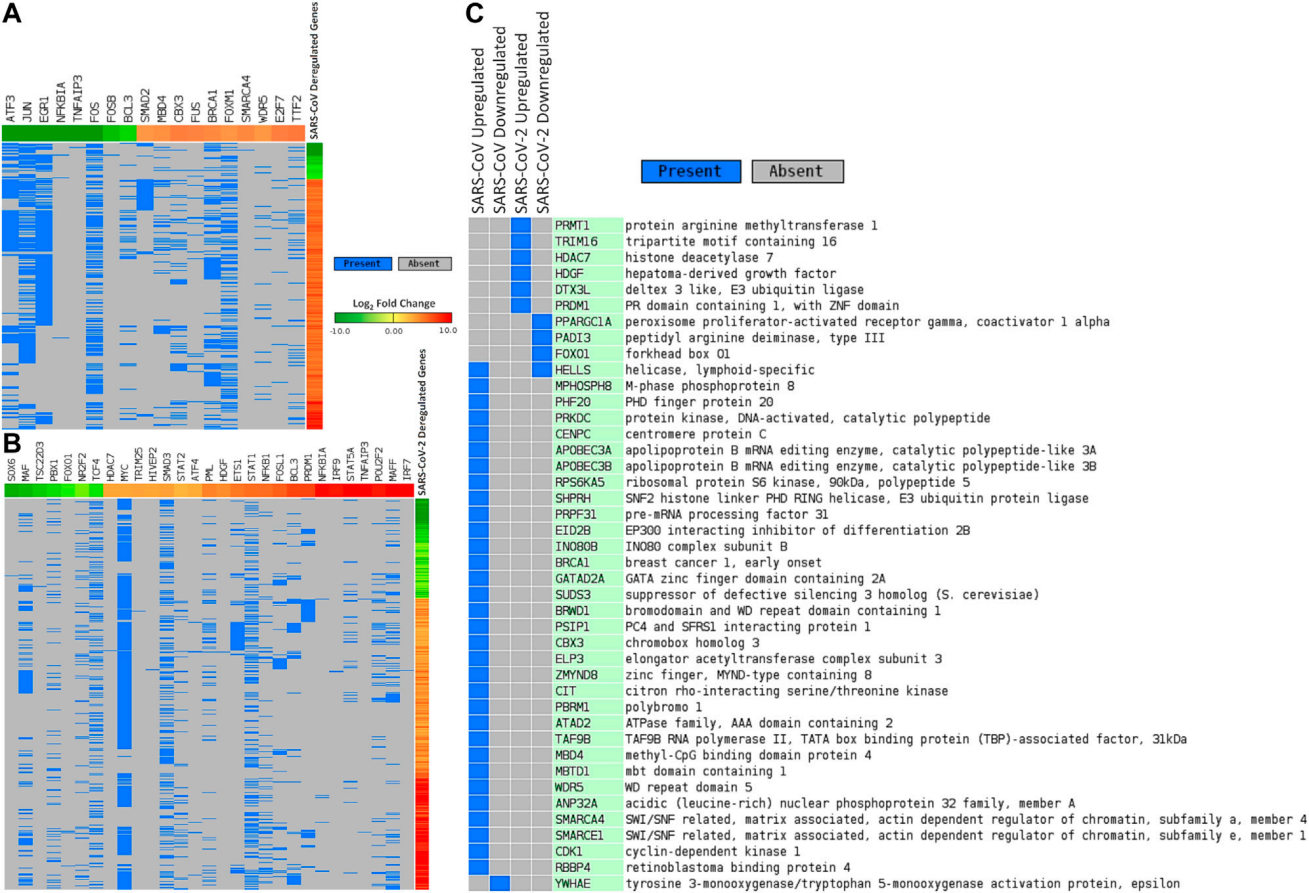
Differentially expressed genes in **(A)** SARS-CoV and **(B)** SARS-CoV-2 infections and associated deregulated transcription factors that can bind around their promoters. **(C)** Deregulated epigenetic factors in SARS-CoV and SARS-CoV-2 infections. In Figures 4A,B, genes are represented vertically, while the associated transcription factors are shown horizontally. Expression values of the TFs/genes are shown in Log_2_ fold change scale for both. Color toward red indicates upregulation and color toward green indicates downregulation. Blue color indicates presence and gray color indicates the absence of a term.

We sought to find out the TFs that have promoter binding preferences for differentially expressed genes in SARS-CoV and SARS-CoV-2 infections. To achieve this, we utilized CistromeDB (Zheng et al., 2018). We identified 18 and 29 such TFs overrepresented around differentially expressed genes of SARS-CoV and SARS-CoV-2, respectively (Figures 4A,B, Supplementary Figure S3). Among those TFs, only 3 (NFKB1A, TNFAIP3, and BCL3) were common for deregulated genes of both infections. Nineteen of 29 TFs, which were overrepresented for SARS-CoV-2 deregulated genes, were also upregulated upon SARS-CoV-2 infections (data not shown).

### Downregulated Genes in SARS-CoV-2 Infection Can Be Targeted by Different Human miRNAs

Viral infection leading to the expression of different host miRNAs is a common phenomenon (Bruscella et al., 2017). To elucidate such roles of the human miRNAs in SARS-CoV and SARS-CoV-2 infections, we considered those miRNAs from miRTarBase database (Huang et al., 2019a) that can particularly target the downregulated genes in these infections. We detected 13 and 389 candidate miRNAs targeting 17 and 123 downregulated genes in SARS-CoV-infection and SARS-CoV-2-infection, respectively (Supplementary Material S3). Among those, only 7 miRNAs (hsa-miR-148a-3p, hsa-miR-146a-5p, hsa-miR-155-5p, hsa-miR-146b-5p, hsa-miR-27a-3p, hsa-miR-146b-3p, and hsa-miR-141-5p) were observed to be targeting the downregulated genes in both infections.

### Upregulated Transcription Factors in SARS-CoV-2 Infection Modulate Different Host miRNAs

To detect the upregulated TFs that can preferentially bind around the promoters of the host miRNAs and their associated functional roles, we have utilized the TransmiR v2.0 database (Tong et al., 2019). We obtained 5 and 14 such upregulated TFs that might have a regulatory role in 14 and 90 host miRNAs in SARS-CoV and SARS-CoV-2 infections, respectively (Supplementary Material S4). Though these TFs were completely different in both infections, we have found 6 host miRNAs (hsa-miR-146a, hsa-miR-146b, hsa-miR-155, hsa-miR-141, hsa-miR-200a, and hsa-miR-27a) that were commonly modulated by TFs in both infections. Intriguingly in SARS-CoV-2 infection, we noticed 2 host miRNAs (hsa-miR-429 and hsa-miR-1286) that were influenced by the upregulated TF NFKB1 and have associations with several downregulated genes (*BCL2L11*, *FKBP5*, and *TP53INP1* for hsa-miR-429; *CLU* for hsa-miR-1286).

### Several Host Epigenetic Factors Can Modulate the Deregulation of Gene Expression in SARS-CoV-2 Infection

Next, we pursued the epigenetic factors that are deregulated and tried to elucidate if their deregulation could play a role in the overall differential gene expression. In this pursuit, we searched the EpiFactors database (Medvedeva et al., 2015) and identified 33 and 10 epigenetic factors that were deregulated in SARS-CoV and SARS-CoV-2 infections, respectively (Figure 4C). Among the 10 factors found in SARS-CoV-2 infection, 6 (*PRMT1*, *TRIM16*, *HDAC7*, *HDGF*, *DTX3L*, and *PRDM1*) were upregulated and 4 (*PPARGC1A*, *PADI3*, *FOXO1*, and *HELLS*) were downregulated.

### Putative Roles of Viral Proteins in Immune Evasion and Disease Pathophysiology of COVID-19 Are Evident From Various Signaling Pathways

Although there are some similarities between SARS-CoV and SARS-CoV-2 genetic architecture, it is yet to be known whether they modulate common host pathways or not. Also, it is largely unknown how SARS-CoV-2 exhibits some unique clinical features, despite sharing many similarities, in terms of viral genes, with SARS-CoV.

As now the probable genetic and epigenetic regulators behind the differential gene expression have been identified, we aimed to explore how these deregulated genes are playing a role in the battle between virus and host. To obtain a detailed view of the outcomes resulting from viral-host interactions and how SARS-CoV-2 uses its proteins to evade host innate immune response, we mapped the significantly deregulated genes and host interacting proteins in different overrepresented functional pathways using KEGG mapper (Kanehisa and Sato, 2020). Analyzing the pathways, we modeled several host-virus interactions in signaling pathways leading to the ultimate viral immune escape mechanisms. SARS-CoV-2 can blockade several signaling pathways such as HIF-1 signaling, autophagy, RIG-I signaling, receptor-interacting protein kinase 1- (RIP1-) mediated signaling, beta-adrenergic receptor signaling, insulin signaling, fatty acid oxidation and degradation pathway, IL-17 signaling, Toll-like receptor signaling, phagosome formation, arachidonic acid metabolism, and PVR signaling. Aberration of these pathways might give SARS-CoV-2 a competitive edge over the host immune response. Also, SARS-CoV-2 can prevent the relaxin downstream signaling that plays a crucial role in the lung’s overall functionality and its abnormal regulation might result in the respiratory complications found in COVID-19.

From previous studies, we compiled the information on deregulated genes (Blanco-Melo et al., 2020) and virus-host interactome (Gordon et al., 2020) in SARS-CoV-2 infection to get detailed pictures of the affected pathways, which are still obscure. Furthermore, we investigated how our identified host genetic and epigenetic factors are playing a role in these pathways. Taking a closer look, we illustrated some pathways that SARS-CoV-2 might be using but not SARS-CoV.

#### SARS-CoV-2 Host Interactions Might Lead to Respiratory Complications in COVID-19

**Figure 5:**
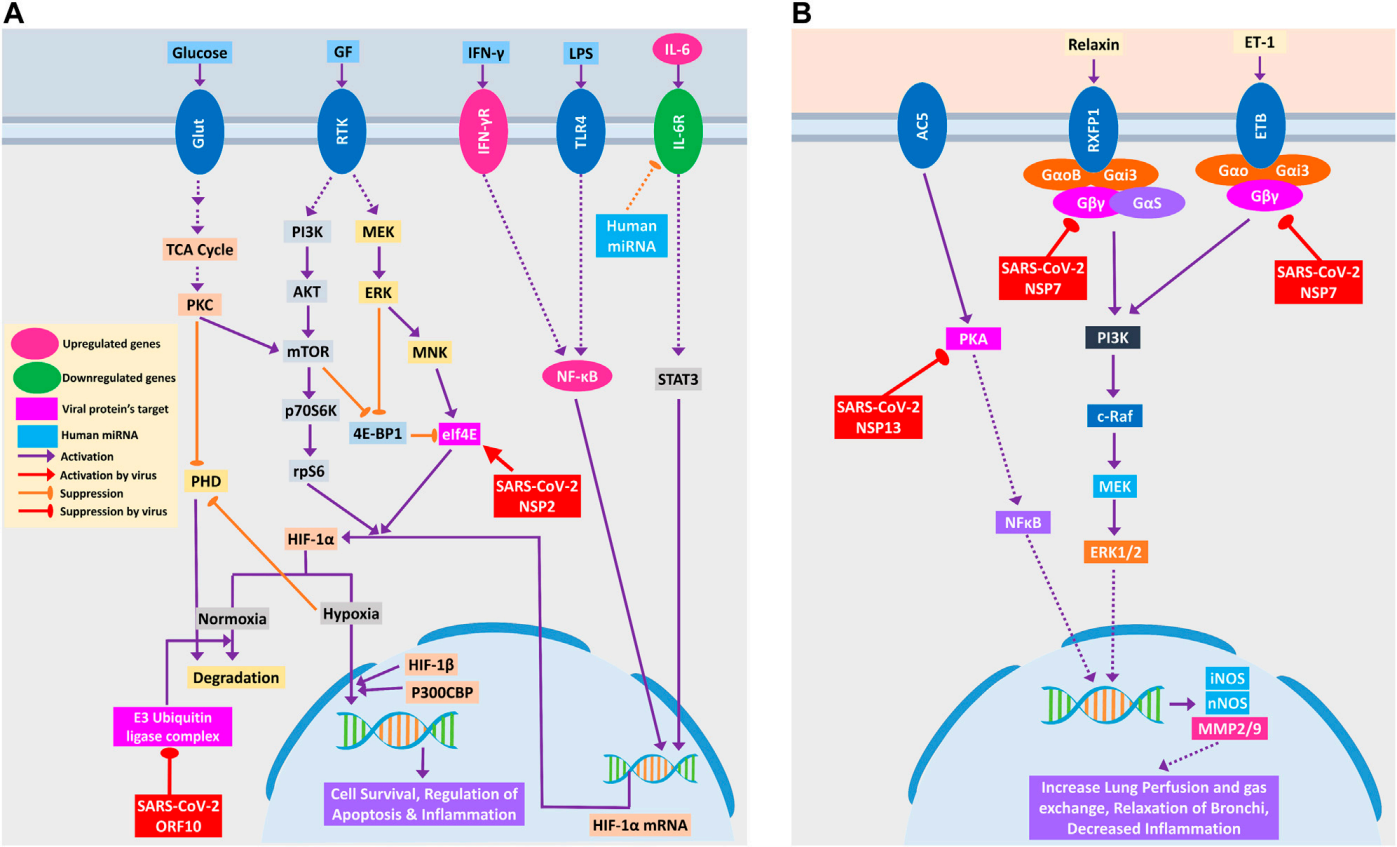
**(A)** HIF-1 signaling pathway and **(B)** relaxin signaling pathway in lungs. Upregulated genes are in pink color, while downregulated genes are in green color. Magenta and light blue represent viral protein’s target and host miRNAs, respectively. Violet pointed arrows indicate activation, while orange blunt arrows indicate suppression. Red pointed and blunt arrows indicate activation and suppression by virus, respectively.

COVID-19 patients are reported to have suffered from hypoxic conditions due to breathing complications (Cascella et al., 2020). HIF-1 signaling pathway provides significant support mechanisms during this hypoxic condition by activating a wide range of other stress-coping mechanisms and ultimately leads to the survival of the stressed cells (Chen and Sang, 2016). So, if the infected cells utilize this survival mechanism, the viruses propagating within these will also be saved. Thus, SARS-CoV-2 could be supporting these survival mechanisms, as ORF10 protein binds and inhibits the E3 ubiquitin ligase complex, which degrades HIF-1α protein (Figure 5A); a similar phenomenon was also evident in other viruses (Mahon et al., 2014). Additionally, it can also stimulate the functions of eIF4E (Eukaryotic Translation Initiation Factor 4E) for the overproduction of HIF-1α (Figure 5A); a similar function was recorded for sapovirus protein VPg (Montero et al., 2015).

SARS-CoV-2-mediated overexpression of HIF signaling products might lead to pulmonary hypertension and acute lung injury (Shimoda and Semenza, 2011), which can be correlated with frequent lung failure in critically infected patients. HIF signaling promotes hypoxia-induced endothelial cell proliferation (Krock et al., 2011), which in turn might lead to aberrant clot formation in the presence of amplified inflammation (Yau et al., 2015) that is frequently found in many COVID-19 patients (Rotzinger et al., 2020).

Relaxin signaling plays a significant role in maintaining the lung’s overall functionality by maintaining lung perfusion and gas exchange, relaxation of bronchi, and decreased inflammation in the lungs (Alexiou et al., 2013). SARS-CoV-2 NSP7 protein can perturb this signaling by binding and inhibiting the relaxin receptors and can prevent the production of NOS and MMP2/9 through PI3K to ERK1/2 axis (Figure 5B). NSP13, another SARS-CoV-2 protein, might bind and block PKA, and its failure to activate NFκB may lead to the blockade of the whole relaxin signaling pathway (Figure 5B). Aberration of this signaling pathway by SARS-CoV-2 possibly leads to breathing complications in COVID-19 patients.

**Figure 6:**
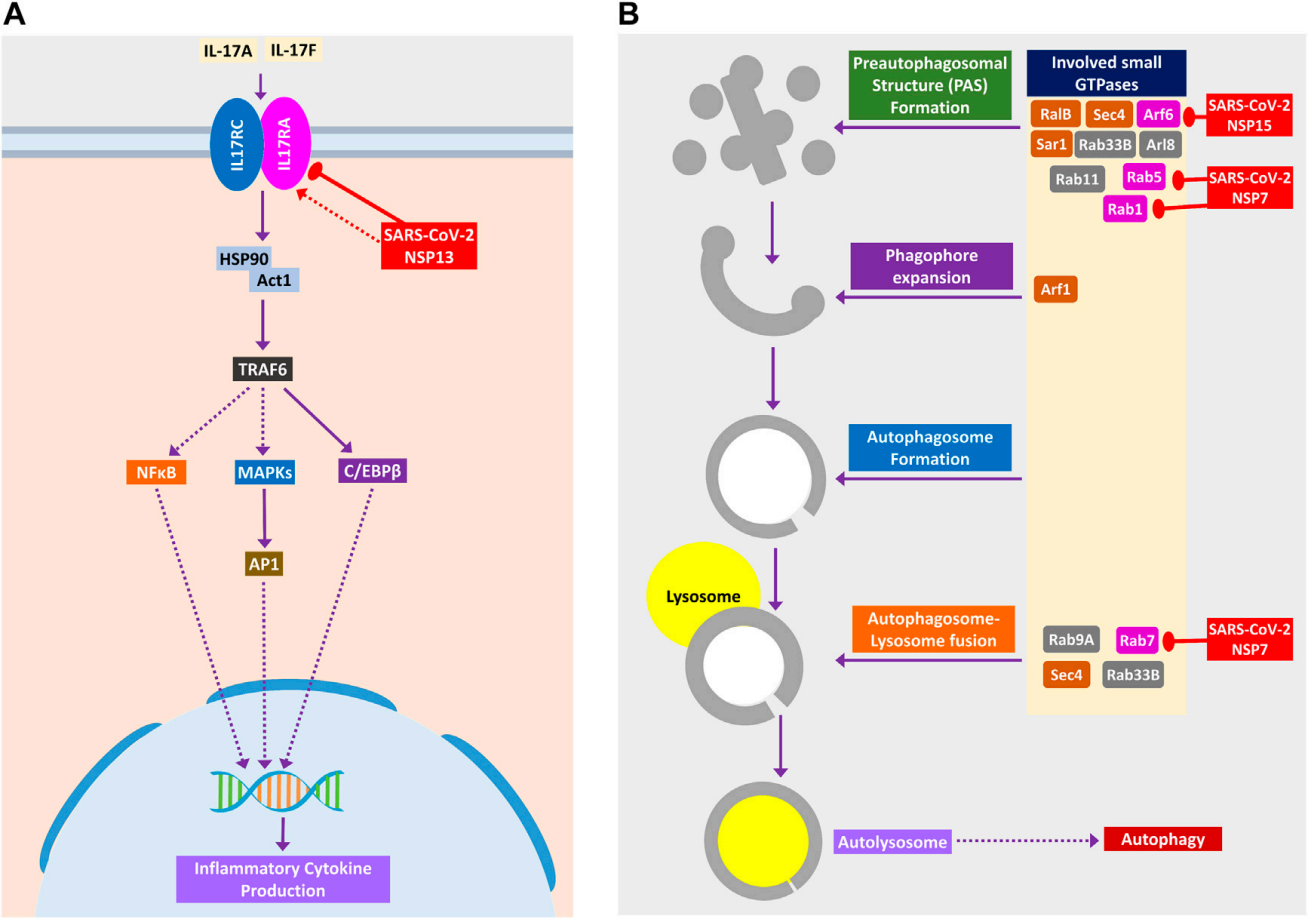
**(A)** IL-17 signaling pathway. **(B)** Small GTPases in phagosome formation and maturation. Color codes are as in Figure 5.

Binding of IL-17 receptor by SARS-CoV-2 NSP13 might increase the downstream signaling by activated TRAF6 to NFκB/MAPKs/CEBPB, which might cause some pathogenic inflammatory responses (Figure 6A). Though IL-17 signaling is initially helpful in the host defense, still its aberrant expression might lead to pathogenic inflammatory responses leading to lung complications such as chronic obstructive pulmonary disease (COPD), lung fibrosis, pneumonia, and acute lung injury (Gurczynski and Moore, 2018).

#### SARS-CoV-2 Might Impede Autophagy and Phagocytosis to Ensure its Existence

**Figure 7:**
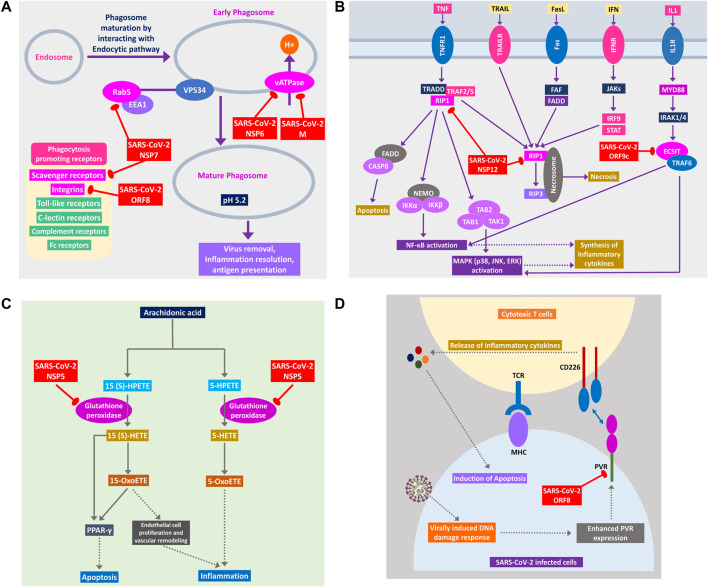
**(A)** Phagosome maturation pathway, **(B)** RIP1 and ECSIT signaling pathway, **(C)** arachidonic acid metabolism pathway, and **(D)** cytotoxic T cells signaling pathway using CD226 and PVR axis. Color codes are as in Figure 5.

During viral infection, one important immune response is autophagy that destroys the virus-infected cells, and viruses are often found to modulate it for their survival (Ahmad et al., 2018). SARS-CoV-2 can inhibit the formation of autophagosome (Figure 6B) and phagosome (Figure 7A) using several of its proteins. SARS-CoV-2 NSP15 and NSP7 might bind and inhibit the small members of Rab, Arf GTPases family (Figure 6B) necessary for autophagosome formation (Bento et al., 2013). SARS-CoV-2 NSP7 and ORF8 can inhibit several phagocytoses, promoting receptors such as scavenger receptors and integrins (Figure 7A); SARS-CoV-2 NSP6 and M protein might block v-ATPase, thus preventing the lowering of pH inside phagosome and preventing its maturation (Figure 7A); SARS-CoV-2 NSP7 prevents the phagosome-endosome/lysosome fusion by targeting Rab5 GTPase, which might result in viral pneumonia (Jakab et al., 1980) (Figure 7A).

#### SARS-CoV-2 Can Hinder Host Apoptotic Responses Necessary for Its Growth

Apoptosis plays important intracellular host immune response to reduce the further spread of viruses from the infected cells (Barber, 2001). Several signaling pathways are involved in eliciting this apoptotic response inside the infected cells, which can be suppressed by the viral proteins, as follows. NSP12 is found to target RIP1; thus, it might fail to relay signaling to CASP8/FADD-mediated apoptosis and necrosis by RIP1/RIP3 complex (Figure 7B). NSP5 might block glutathione peroxidase that is involved in 15(S)-HETE production and ultimately 15-oxoETE production (Figure 7C); thus, apoptotic induction by these metabolites through PPARγ signaling axis might not take place (Powell and Rokach, 2015). ORF8 can block PVR-CD226 signaling in cytotoxic T cell–mediated apoptosis (Figure 7D), as many viruses were reported to block PVR from expressing in the infected cell’s membranes (Cifaldi et al., 2019). Also, infection-induced host miRNAs in β2-adrenergic signaling (Supplementary Figure S4) and insulin signaling can block apoptosis of the infected cell.

#### Host Antiviral Inflammatory Cytokine and Interferon Production Pathways Could Be Perturbed by SARS-CoV-2 Proteins

Cytokine signaling pathways play a major role in suppressing viral infections (Mogensen and Paludan, 2001). Similar inflammatory cytokine production pathways were also reported in human coronaviral infections (Fung and Liu, 2019). We observed that SARS-CoV-2 proteins are interacting with the members of these pathways, and this might alter the signaling outcomes of these pathways to reduce the overall production of virus infection–induced inflammatory cytokines.

**Figure 8:**
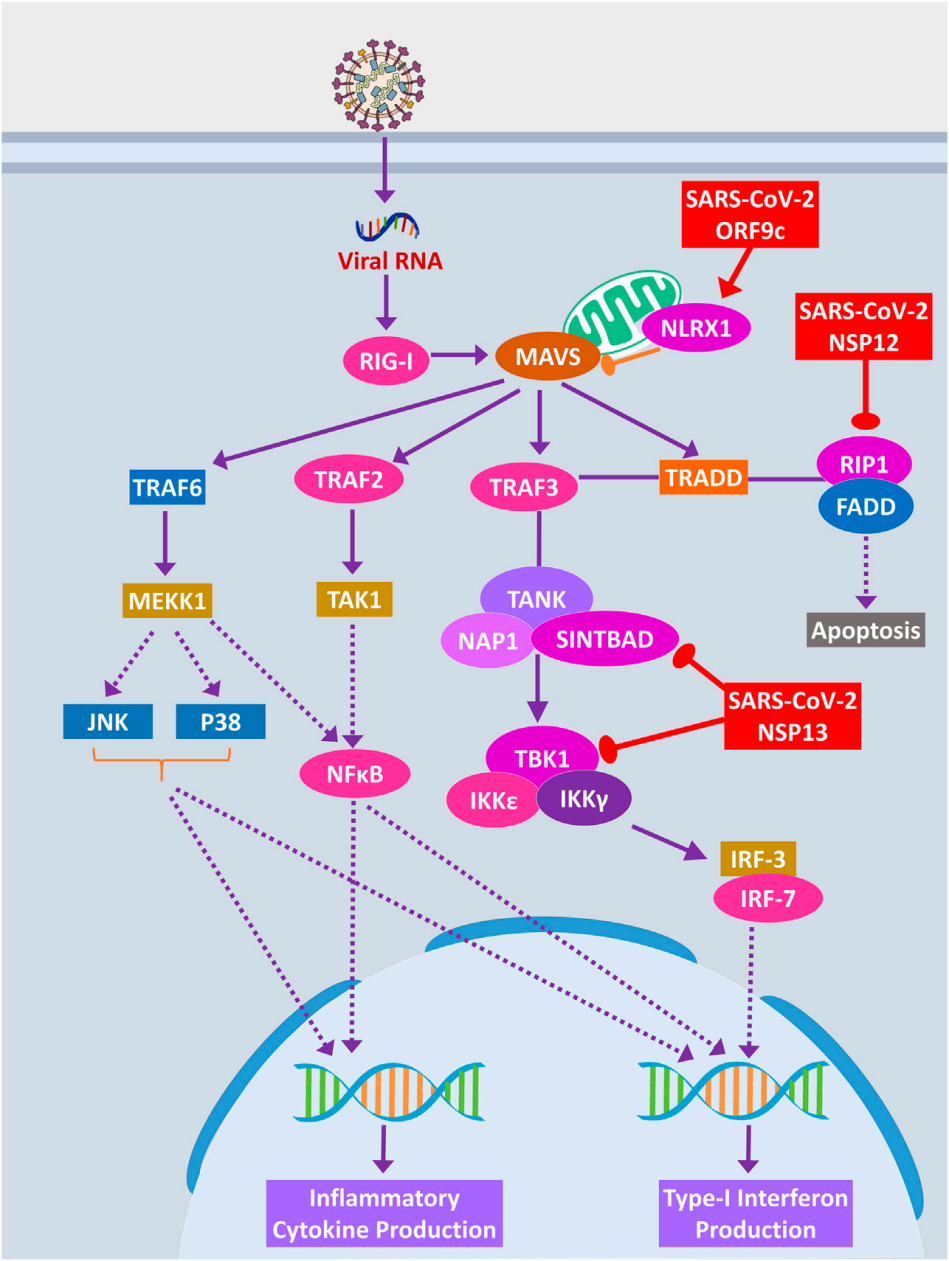
RIG-I signaling pathway. Color codes are as in Figure 5.

RIG-I signaling plays an important role in producing antiviral inflammatory cytokines and interferons and induction of apoptosis (Chan and Gack, 2015). SARS-CoV-2 ORF9c protein can activate NLRX1 to degrade MAVS, which results in failure of inflammatory cytokine production by NFkB via TRAF2/TAK1 or TRAF6/MEKK1 pathways (Figure 8). NLRX1 activity was previously found to be upregulated by HCV infection (Qin et al., 2017). By binding TBK1 and SINTBAD, SARS-CoV-2 NSP13 might inhibit the interferon production by IRF3/IRF7 stimulation (Figure 8).

A previous study suggested that RIP1 signaling plays a key role in human coronavirus infection (Meessen-Pinard et al., 2017). RIP1 along with TRADD and TRAF2/5 activates NFκB and MAPKs, then inducing the production inflammatory cytokines; this signaling axis might be blocked by SARS-CoV-2 NSP12 protein-RIP1 interaction, therefore inhibiting the antiviral mechanisms exerted by this signaling pathway (Figure 7B). SARS-CoV-2 ORF9c can also inhibit the ECSIT/TRAF6 signaling axis (Figure 7B), which plays pivotal antiviral roles by activating NFκB and MAPKs signaling (Lei et al., 2015).

β2-Adrenergic signaling plays important antiviral roles in respiratory virus infections (Ağaç et al., 2018). SARS-CoV-2 NSP13 interacts with PKA, which might inhibit the activation of CREB by PKA for producing antiviral inflammatory responses (Supplementary Figure S4).

Previous studies showed that arachidonic acids suppress the replication of HCoV-229E and MERS-CoV (Yan et al., 2019). 15-OxoETE, an arachidonic acid metabolism product that promotes pulmonary artery endothelial cell proliferation during hypoxia (Ma et al., 2014), in turn results in vascular remodeling and leakage of inflammatory cytokines (Powell and Rokach, 2015). 5-OxoETE, another arachidonic acid metabolism product that can also induce inflammation (Powell and Rokach, 2015), production of both compounds might be hindered by SARS-CoV-2 NSP5 as it interacts with an upstream metabolic enzyme glutathione peroxidase (Figure 7C).

Previously, it was reported that IL-17 signaling enhances antiviral immune responses (Ma et al., 2019). SARS-CoV-2 NSP13 can bind IL-17 receptor and inhibit the downstream signaling from IL-17 receptor to TRAF6 for activating NFκB/MAPKs/CEBPB signaling axis, thus decreasing the antiviral inflammatory responses (Figure 6A).

**Figure 9:**
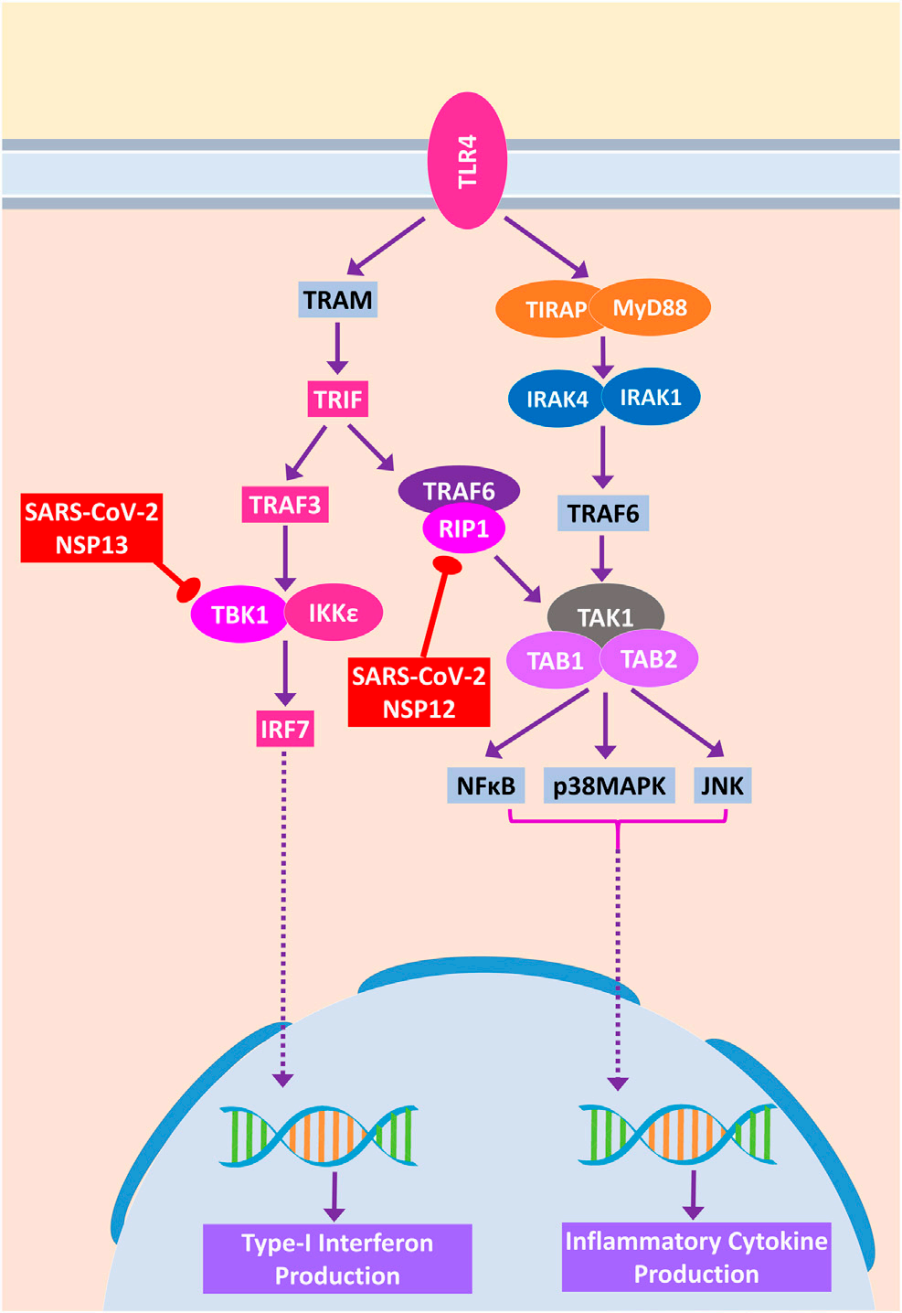
Toll-like receptor 4 (TLR4) signaling. Color codes are as in Figure 5.

During acute viral infections, Toll-like receptor 4 (TLR4) signaling plays an important role in eliciting inflammatory responses (Olejnik et al., 2018). SARS-CoV-2 protein NSP13 interacts with TBK1, which might reduce the signaling from IRF7, resulting in less IFN-I productions, whereas NSP12 interacts with RIP1; as a result, the activation of downstream NFκB and MAPKs (p38 and JNK) pathways and induction of inflammatory responses from these pathways might be stalled (Figure 9).

#### SARS-CoV-2 Infection Can Negatively Regulate Fatty Acid Metabolism for Its Proliferation

**Figure 10:**
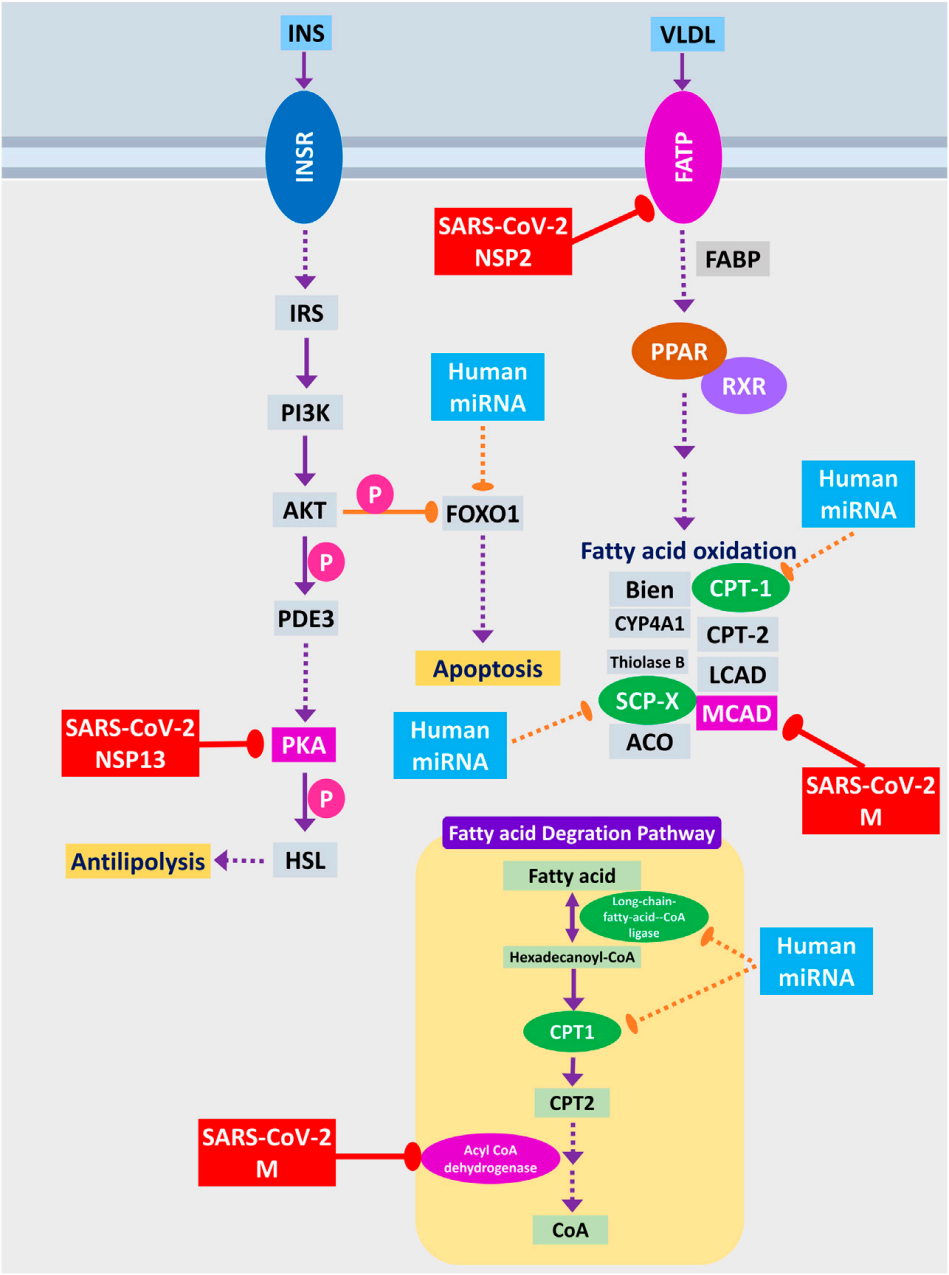
Fatty acid oxidation, fatty acid degradation, and antilipolysis through insulin signaling. Color codes are as in Figure 5.

Host lipid and fatty acid metabolism play a crucial role in maintaining the viral life cycle and propagation inside the infected cells as viruses tend to utilize host metabolic pathways to their aid (Martín-Acebes et al., 2012). Cui et al. (2019) showed that impairment of fatty acid oxidation could lead to acute lung injury (Cui et al., 2019). SARS-CoV-2 NSP2 can interact with FATP receptor of fatty acid oxidation pathway, whereas M protein can interact and destabilize MCAD of fatty acid oxidation pathway; moreover, two other members can be the targets of human miRNA (Figure 10). MCAD deficiency leads to pulmonary hemorrhage and cardiac dysfunction in neonates (Maclean et al., 2005). So, this destabilization might lead to acute lung injury during COVID-19. Increased fatty acid biosynthetic pathways are found in several viral infections for their efficient multiplications (Martín-Acebes et al., 2012), so it is logical for SARS-CoV-2 to inhibit the fatty acid degrading pathways. SARS-CoV-2 NSP13 protein was found to interact with insulin signaling–mediated antilipolysis by PKA-HSL signaling axis; SARS-CoV-2 M and several host miRNAs can inhibit fatty acid degradation to CoA through CPT1-CPT2 metabolic axis (Figure 10) so that more fatty acids can be produced.

## Discussion

The tug-of-war between the viral pathogens and the infected host’s response upon the infection is a critical and complex relationship deciding the ultimate fate of infection. Although most of the time, successful removal of the virus is achieved through host immune response, viruses have also evolved some immune evasion mechanisms to escape the immune surveillance of the host, thus making the outcomes of the disease more complicated (Fung et al., 2020). Similar interactions were also found in other human coronaviruses that modulate the host immune responses (Fung and Liu, 2019; Fung et al., 2020). In this study, we have depicted how SARS-CoV-2 and host protein interactome lead to the probable immune escape mechanisms of this novel virus, along with the functional roles of other host epigenetic factors in this interaction as host epigenetic factors serve important roles in viral infections (Bussfeld et al., 1997; Paschos and Allday, 2010; Girardi et al., 2018).

Our analysis showed several TFs are capable of binding around the promoters of deregulated genes found in SARS-CoV-2 infection, which were absent in SARS-CoV infection. Some of these downregulated TFs in SARS-CoV-2 infection, such as MAF and FOXO1, can elicit proviral responses (Kim and Seed, 2010; Lei et al., 2013). Also, some upregulated TFs in SARS-CoV-2 infection, such as HDAC7 (Herbein and Wendling, 2010), STAT2 (Le-Trilling et al., 2018), ATF4 (Caselli et al., 2012), and FOSL1 (Cai et al., 2017), can facilitate the progression of the viral life cycle and immune evasion in the host. However, other upregulated TFs in SARS-CoV-2 infection TRIM25 (Martín-Vicente et al., 2017), SMAD3 (Qing et al., 2004), STAT1, IRF7, and IRF9 (Chiang and Liu, 2019) might play a role in the antiviral immunity upon infection.

Interestingly, we detected 2 miRNAs, namely, hsa-miR-429 and hsa-miR-1286, whose associated TFs were upregulated and their target genes were downregulated, suggesting they might have some roles in this host-virus interaction. In RSV infection, hsa-miR-429 was found to be upregulated in severe disease conditions (Inchley et al., 2015). Other studies showed that this miRNA plays an important role in promoting viral replication and reactivation from latency (Ellis-Connell et al., 2010; Bernier and Sagan, 2018). So, the expression of this miRNA in SARS-CoV-2 infection can lead to similar disease outcomes.

Upregulated epigenetic factors in SARS-CoV-2 infections can be both a boon and a bane for the host, as factors such as TRIM16 (van Tol et al., 2017) and DTX3L (Zhang et al., 2015) can provide antiviral responses, whereas factors such as PRDM1 (also known as BLIMP-1) (Lu et al., 2014) and HDAC7 (Herbein and Wendling, 2010) can act as proviral factors.

Upregulated TFs such as SMAD3, MYC, NFKB1, and STAT1 might be involved in the upregulation of hsa-miR-18a, hsa-miR-155, hsa-miR-210, hsa-miR-429, and hsa-miR-433, which can, in turn, downregulate the HIF-1 production (Supplementary Figure S5) (Serocki et al., 2018). Also, epigenetic factors like PRMT1 can downregulate HIF-1 expression, whereas factors like HDAC7 can increase the transcription of HIF-1 (Luo and Wang, 2018). We found that when MYC, SMAD3, and TNF TFs are upregulated, they can activate autophagy and apoptosis promoting miRNAs such as hsa-miR-17, hsa-miR-20, and hsa-miR-106 (Supplementary Figure S5) (Xu et al., 2012). RIG-I signaling can induce miRNAs such as hsa-miR-24, hsa-miR-32, hsa-miR-125, and hsa-miR-150 to neutralize viral threats (Li and Shi, 2013), and we also found that these miRNAs can be transcribed by the upregulated TFs (Supplementary Figure S5). RIP1 can be targeted by induced hsa-miR-24 and hsa-miR-155 (Liu et al., 2011; Tan et al., 2018) in SARS-CoV-2 infection (Supplementary Figure S5). IL-17F can be targeted by the upregulated hsa-miR-106a, hsa-miR-17, and hsa-miR-20a, whereas IL-17A expression can be modulated by the upregulated hsa-miR-146a and hsa-miR-30c (Supplementary Figure S5) (Mai et al., 2012). We also found that different upregulated TFs induced miRNAs such as hsa-miR-146a and hsa-miR-155 can downregulate TLR4 signaling (Supplementary Figure S5) (Yang et al., 2011).

From the enrichment analysis, we found that deregulated genes were also involved in processes and functions such as heart development, kidney development, AGE-RAGE signaling pathway in diabetic complications, zinc ion binding, and calcium ion binding (Figures 2A,B). Impairment of these organ-specific functions might suggest the increased susceptibility to COVID-19 in patients having comorbidities. Zinc and calcium ions play significant roles in activating different immune responses; aberrant regulation of these might be lethal for COVID-19 patients (Verma et al., 2011; Read et al., 2019).

Host antiviral immune responses encompassing different mechanisms such as autophagy, apoptosis, interferon signaling, and inflammation play a fundamental role in neutralizing viral threats found not only in human coronaviruses (Fung and Liu, 2019) but also in other viral infections (Barber, 2001; Mogensen and Paludan, 2001; Ahmad et al., 2018). Viruses have also evolved hijacking mechanisms to bypass all those mechanisms for their survival (Fung et al., 2020). From our analyses, we have observed that SARS-CoV-2 might have similar modes of action for its successful immune escape from the host immune surveillance. While all these mechanisms are supporting viral propagation, the host suffers their adverse effects, resulting in severe complications in COVID-19 patients.

All of these findings suggest that a very complex host-virus interaction takes place during the SARS-CoV-2 infections. During the infections, while some host responses have a significant impact on eradicating the viruses from the body, the virus modulates some critical host proteins and epigenetic machinery for its successful replication and evasion of host immune responses. Due to these complex cross-talk between the host and virus, the disease complications of COVID-19 might arise.

In this present study, our goal was to model putative pathogenic alterations within the host biological pathways, and for simplicity, we used only one infection model each for SARS-CoV and SARS-CoV-2. We used several transcriptomes and interactome data from various infection systems to formulize the modeling. Though this modeling could be more precisely done if there were data on uniform infection system, such approach is frequently used in computational systems biology–related modeling approaches when the required data are scarce. We integrated epigenetic factors and miRNAs into our modeling based on the DE patterns observed from the infection models, as the direct evidence of their modulation is yet to be experimentally reported in SARS-CoV-2 infection. Our results would be helpful for further in-depth experimental design to understand the detailed molecular mechanisms of COVID-19 pathogenesis and to develop some potential therapeutic approaches targeting these host-virus interactions.

## Data Availability Statement

The original contributions presented in the study are included in the article/Supplementary Material; further inquiries can be directed to the corresponding author.

## Author Contributions

AI and MK conceived the project, designed the workflow, and performed the analyses. MK and AI wrote the manuscript. Both authors read and approved the final manuscript.

## Conflict of Interest

The authors declare that the research was conducted in the absence of any commercial or financial relationships that could be construed as a potential conflict of interest.
